# Comparative study of the removal of urea by electrocoagulation and electrocoagulation combined with chemical coagulation in aqueous effluents

**DOI:** 10.1038/s41598-024-81422-x

**Published:** 2024-12-23

**Authors:** A. Shaban, M. E. Basiouny, Osama A. AboSiada

**Affiliations:** 1 Civil Engineering Department, Benha Faculty of Engineering, Benha, 13512 Egypt; 2 El Arish Faculty of Engineering, Sinai University, El-Arish, 45511 Egypt

**Keywords:** Urea removal, Chemical coagulation, Electrocoagulation, Organic pollutants, Sequential electrocoagulation, Chemical coagulation, Environmental impact, Civil engineering

## Abstract

Urea is a major issue in human wastewater because it may be easily broken down by the urease enzyme produced by bacteria, leading to ammonia production. Due to its ability to increase soil pH and eutrophicate streams, ammonia-containing effluent emissions pose environmental and health risks. This study aimed to evaluate the effectiveness of various treatment approaches in reducing urea concentrations by comparing the removal rates of conducting electrocoagulation (EC), EC followed by chemical coagulation (EC-CC), and CC followed by electrocoagulation (EC-CC). Numerous electrocoagulation parameters have been investigated, including current density, electrode gap distance, electrolyte type, concentration, and electrolysis duration. The electrode morphology was examined using a scanning electron microscope, while the produced sludge was analyzed using Fourier transform infrared spectroscopy. Three kinds of aluminum coagulants—potash alum, aluminum sulfate, and aluminum chloride—were used in the chemical coagulation, while the electrocoagulation was optimized at 30 A/m^2^. The results of this investigation suggest that the application of EC-CC, regardless of the type of coagulant used in both synthetic and real effluent, could marginally improve the efficacy of urea removal. Conversely, CC-EC exhibits an adverse effect on the efficiency of urea removal in both synthetic and real wastewater. The application of CC-EC demonstrated a significant improvement in the effectiveness of COD removal from actual wastewater, according to experimental results. The study emphasized the effectiveness and economic advantages of electrocoagulation over EC-CC and CC-EC techniques, used to remove urea from both real and synthetic wastewater.

## Introduction

Significant amounts of urea are produced due to its widespread use. Leachate from agricultural fields and farms, human effluent, and the utilization of urea as a raw material in industries and plants all contribute to its presence in the environment^1^. Considering the average daily urine output of an adult (1.5 L) and the urea concentration of approximately 22–23 g/L, it is evident that domestic effluent containing human urine is significantly enriched in urea^2^. In addition, agricultural fertilizer urea gains infiltration into waterways, predominantly via runoffs, and ultimately reaches receiving bodies of water^3^. Urea is a significant issue in human wastewater because it can undergo hydrolysis by the urease enzyme produced by microorganisms, leading to ammonia formation. Ammonia effluents raise soil pH and eutrophicate streams, harming both the environment and humans. Additionally, the atmosphere can receive free ammonia, which can lead to the production of nitrates and ammonium sulfates^4^. The imperative to diminish the urea concentration in wastewater plants has grown substantially since the early 1970s, in part as a result of more stringent environmental regulations. Beginning in the twenty-first century, most regulations stipulated a maximum concentration of 10 ppm, whereas in the 1990s, 100 ppm was deemed permissible^5^. A wide range of methodologies have been implemented to remove urea, including adsorption, enzymatic hydrolysis, thermal hydrolysis, chemical and electrochemical oxidation, biological treatment, and membrane separation^1,6^. While thermal hydrolysis has shown significant efficacy in removing urea, its implementation is associated with substantial expenses. Biological treatment achieves a high rate of urea removal, but it generates ammonia, requiring subsequent treatment. Additional methodologies, including enzymatic hydrolysis, adsorption, and membrane separation, have demonstrated urea removal rates between moderate and low^6^. It has also been demonstrated that the electrochemical approach is a viable technique for the removal of urea from both synthetic and real wastewater. Under mild operating conditions, urea can be oxidized, and the primary byproducts of the decomposition are innocuous gases, namely N_2_ and CO_2_^4,7^.

Electrocoagulation (EC) is a method that involves creating an electric field in the effluent to destabilize dissolved and suspended matters in a liquid media by creating active coagulants. Both chemical and physical mechanisms are used in electrocoagulation to remove pollutants^8,9^. It combines the advantageous aspects of coagulation, flotation, and electrochemistry^8^. EC was utilized to remove a variety of contaminants, including pharmaceuticals, heavy metals, suspended particles, and organic and inorganic pollutants^10,11^. EC systems are capable of oxidizing organic matter via chlorine formation at the anode; nevertheless, the extent of chlorine formation is contingent upon the initial sodium chloride concentration^12^. An EC unit in its most basic form comprises two electrodes that are externally linked to a power source and submerged in a container containing the aqueous solution required for treatment. EC is characterized by its simple and user-friendly equipment, short operational duration, minimal or absent chemical usage, and reduced sediment production, leading to significant interest and widespread application^13,14^. However, the EC technique has two significant limitations: a high energy demand and the necessity to replace sacrificial anodes upon depletion^15^. The electrocoagulation process is characterized by the following primary processes: (i) anode oxidation, (ii) gas bubbles generation, (iii) floatation and precipitation of the generated flocs. When a current flows through the anodes, oxidation processes at the anode produce cations, while reduction reactions occur at the cathode. The cations formed in oxidation reactions generate metal hydroxides, which contribute to the destabilization of suspended particles. Charge neutralization, adsorption, and sweep coagulation are among the mechanisms that are employed in the EC process^14,16^. The subsequent reactions occur at the electrodes throughout electrocoagulation:

1 $${\text{Al }} \to {\text{ Al}}^{{ + {3}}} + {\text{ 3e}}^{-} {\text{for coagulation }}\left( {{\text{at the anode}}} \right),\;{\text{and}}$$

2 $${\text{3H}}_{{2}} {\text{O }} + {\text{ 3e}}^{-} \to { 3}/{\text{2H}}_{{2}} + {\text{ 3OH}}^{-} \;{\text{for flotation }}\left( {{\text{at the cathode}}} \right)$$

Comparably, urea undergoes the subsequent electro-oxidation reactions:

3 $${\text{CO}}\left( {{\text{NH}}_{2} } \right)_{{2{\text{(aq)}}}} + 6{\text{OH}}^{-}_{{({\text{aq}})}} \to {\text{N}}_{{2({\text{g}})}} + 5{\text{H}}_{2} {\text{O}}_{(1)} + {\text{CO}}_{{2({\text{g}})}} + \, 6{\text{e}}^{-} \left( {{\text{at the anode}}} \right)$$

4 $$6{\text{H}}_{2} {\text{O}}_{{({\text{l}})}} + \, 6{\text{e}}^{-} \to \, 3{\text{H}}_{{2({\text{g}})}} + \, 6{\text{OH}}^{-}_{{({\text{aq}})}} \left( {{\text{at the cathode}}} \right)$$

5 $${\text{CO}}\left( {{\text{NH}}_{{2}} } \right)_{{{2}({\text{aq}})}} + {\text{ H}}_{{2}} {\text{O}}_{{({\text{l}})}} \to {\text{ N}}_{{{2}({\text{g}})}} + {\text{ 3H}}_{{{2}({\text{g}})}} + {\text{ CO}}_{{{2}({\text{g}})}} \left( {{\text{overall}}} \right)$$

Most contemporary research on the electrochemical treatment of urea emphasizes the use of diverse anode materials, such as boron-doped diamond^4^, ruthenium–titanium oxide^17^, nickel^18^, and platinum^19^. Researchers have employed an electrocoagulation technique, utilizing a diverse range of electrode materials, to examine the removal of urea^20,21^. The utilization of copper and iron as anodes to treat synthetic effluent has been demonstrated to remove urea by 40% and 51%, respectively^21^. The urea removal efficiency was demonstrated to be 59% and 30% from synthetic and real wastewater, respectively, when titanium was employed as the anode, according to research conducted by Safwat et al.^20^. Furthermore, an observed urea removal efficiency of 40% and 27% from synthetic and real wastewater was obtained when aluminum was utilized as the anode^20^. Investigations into the electrocoagulation process for urea removal from synthetic and real wastewater demonstrate the necessity for enhancement or integration with additional treatment methods to improve removal efficacy^20–22^. Numerous scientists have incorporated EC into wastewater treatment systems alongside adsorption^23^, chemical coagulation^24^, membrane filtration^25^, and biological treatment^26^, with some of these integrations exhibiting potential energy and cost savings while also improving removal efficiency. The beneficial aspects of both electrocoagulation (EC) and chemical coagulation (CC) procedures are combined in hybrid electrocoagulation-chemical coagulation (EC-CC), an alternate method of treating water. The inherent benefits of integrating EC and CC include a reduction in separation time, enhanced sediment properties (e.g., reduced water content), and a simplification of the dewatering process^15^. Prior research has shown that the cost-effectiveness and efficacy of pollutant removal are enhanced when EC and CC are combined in the remediation of wastewater^15,24^. Our previous work conclusively showed that the combination of CC-EC, utilizing iron electrodes and ferric chloride as the coagulant, is superior to EC alone in effectively removing urea^27^. Consequently, the principal aims of this study are to (1) compare the effectiveness of urea removal via sequential chemical coagulation (CC) and electrocoagulation (EC) with that of conducting electrocoagulation (EC) alone when using aluminum material as electrodes and aluminum salts as coagulants; (2) study the EC under various effective parameters such as current density, electrode gap distance, electrolyte type, electrolyte concentration, and electrolysis time; and (3) analyze the cost of EC at its optimum conditions and compare it with the cost of (EC-CC) and (CC-EC) which are utilized in this research.

## Materials and methods

### An analysis of the properties of the wastewater

For the experiments, both synthetic and real wastewater containing urea were utilized. Sodium chloride (99.50% purity) was utilized as a supporting electrolyte in the majority of the studies using synthetic wastewater, with a concentration of 0.50 g/L, and urea (99% purity) at 1 g/L. Certain investigations employed sodium sulphate as a supporting electrolyte. The real wastewater was collected from Benha wastewater treatment plant, and Table 1 shows its properties. Alum (Potash alum), aluminum sulfate, and aluminum chloride were used in the chemical coagulation process, as well as in the combined electrocoagulation and chemical coagulation.

**Table 1:** Real wastewater characteristics.

Characteristic	Avg. value	Typical range	Unit
pH	7.80	(6.50–8.00)^28^	–
Conductivity	1290	(500–1500)^28^	μS/cm
Urea	793	–	mg/L
COD	560	(200–800)^28^	mg/L
TDS	778	(500–1200)^28^	mg/L

### Experimental setup

**Fig. 1:**
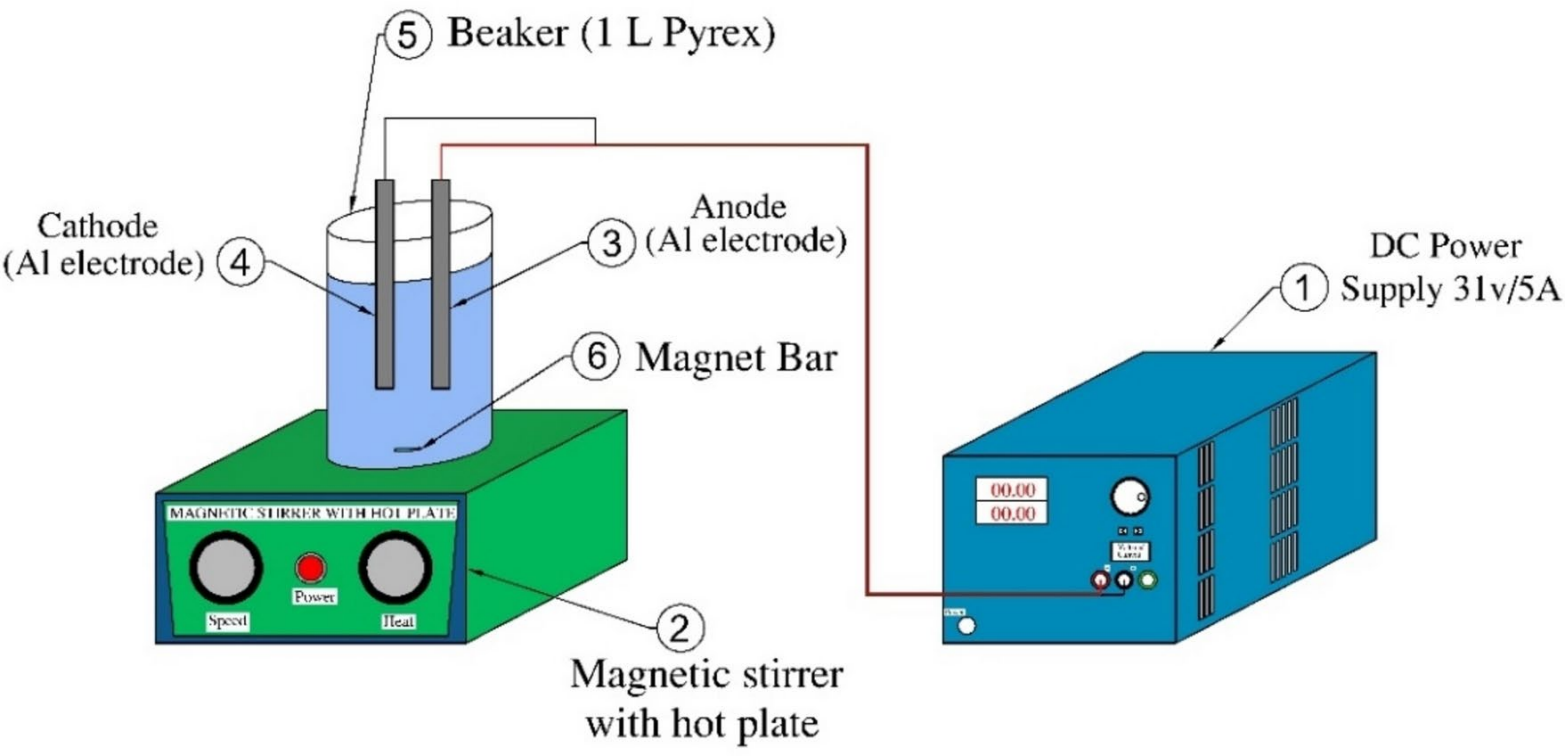
The configuration of electrocoagulation experiment.

**Fig. 2:**
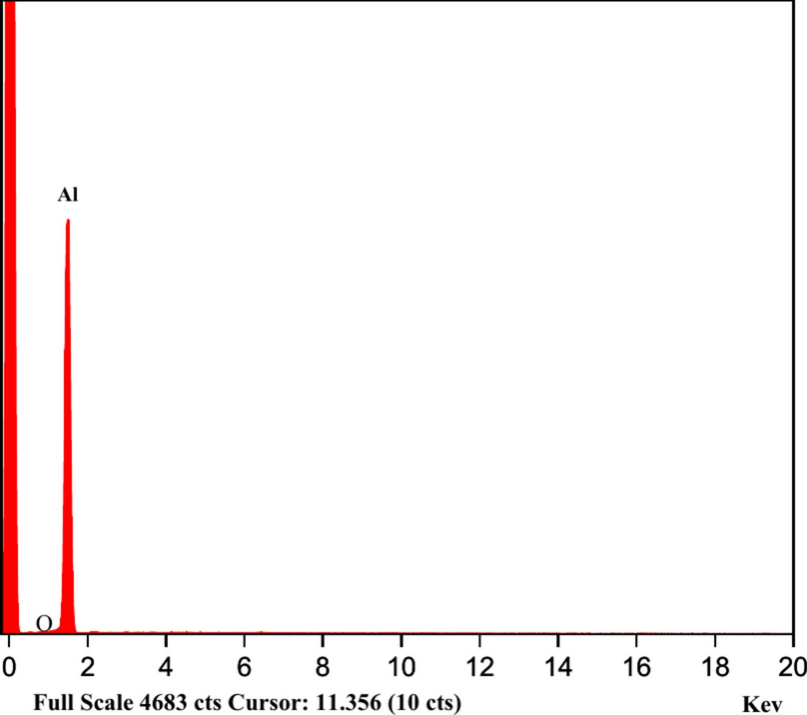
EDX for the aluminum electrode.

As illustrated in Fig. 1, the experimental configuration comprised a 1-L glass beaker positioned with the electrodes in a vertical orientation. The electrodes, composed of aluminum, are linked to a direct-current power source with a voltage of 31 V and a current of 5 A. The anode and the cathode had respective dimensions of 40 mm × 125 mm × 1 mm, and the area of the electrodes’ immersed part was 36 cm^2^. To mitigate floc shearing, an agitating speed of 100 rpm was employed using a magnetic stirrer. The experiment was conducted for 60 min in a batch reactor, with the electrodes positioned at a distance of 4 cm. At 2, 5, 10, 20, 40, and 60 min, samples were collected and filtered to remove any sediment that may have formed throughout the procedure. Prior to each experiment, the electrodes should be cleaned and washed with distilled water to remove any impurities from their surface. The investigation encompassed current density variations, electrode gap distance, electrolyte type, electrolyte concentration, and electrolysis time to determine the optimum conditions for higher efficiency of urea removal. The urea removal process was conducted on a laboratory scale at ambient temperature. As illustrated in Fig. 2, the anode electrode was analyzed utilizing Energy-Dispersive X-ray spectroscopy (EDX).

Comparative analysis was performed on three distinct varieties of aluminum coagulants to determine which one was most efficient at removing urea. The performance of each coagulant was assessed, and the optimal dosage was determined through the implementation of a jar test in this analysis. Potash alum (Aluminum potassium sulfate—AlK(SO_4_)_2_.12H_2_O), aluminum sulfate (Al_2_(SO_4_)_3_), and aluminum chloride Hexahydrate (AlCl_3_.6H_2_O) at concentrations ranging from 0.1 to 0.5 g/L on the removal of urea. The mixing procedure consisted of three stages: an initial period of quick mixing at 125 rpm for 30 s, followed by a subsequent stage of mixing at 75 rpm for a duration of 2 min, and finally, a final stage of mixing at 25 rpm for a duration of 5 min. Subsequent to the mixing procedure, the reactors were allowed to remain undisturbed for 30 min to promote the flocs’ settling^27^. The optimum dose regardless the coagulant type was 0.5 g/L through conducting the jar test. The EC-CC process consisted of repeating the electrocoagulation procedures under ideal conditions and then performing chemical coagulation. The EC was carried out for the subsequent times: 2, 5, 10, 20, 40, and 60 min. Then, 0.5 g/L of one type of all the three coagulants was introduced using the identical chemical coagulation procedure. The identical procedures were followed to complete the CC-EC process; however, the CC operation was performed before the EC procedure.

### Analytical techniques

Conductivity, pH, TDS, and temperature are all measured with a multimeter. HPLC was employed to determine the amount of urea. The method of closed reflux titration was utilized to determine the COD. After any of the removal processes, the percentage of urea and COD removal was estimated using the formula: removal efficiency (%) = $\frac{\text{C}0-\text{Ce}}{\text{C}0}$  × 100. In this case, C_0_ represents the urea or COD concentration in the influent, and C_e_ represents the urea or COD concentration in the effluent. The analyses were carried out with three replicates, and each experiment was conducted twice. The morphology of the electrode was examined using scanning electron microscopy and a Fourier-transform infrared (FTIR) spectrometer to analyze the sediment produced during the procedure.

### Economic factor analysis

Electrode material utilization and the quantity of electrical energy required for the treatment procedure account for the majority of the overall cost associated with EC operations^27,29^. Other costs, such as labour, maintenance, sludge dewatering, and sludge disposal, were deemed to be fixed and consequently excluded from the calculation^24^. Energy usage and electrode material consumption can be computed utilizing Eqs. (6) and (7), respectively.

6 $$\text{Menergy }= \frac{V \times i \times t}{\forall }$$

7 $$\text{Melectrode }= \frac{M \times i \times t}{\forall \times n \times f}$$

where V = average cell voltage (V), *i* = applied current (A), t = electrolysis time (h), ∀ = volume of wastewater in EC units (L), M is the molar mass of the aluminum electrode (26.98 g/mol), n is the number of electrons (3 for aluminum), and F is the Faraday constant (96,485 °C/mol).

To determine the operating costs of the treatment, Eq. (8) was applied.

8 $${\text{Operating cost }} = {\text{ aM}}_{{{\mathrm{energy}}}} + {\text{ bM}}_{{{\mathrm{electrode}}}} + {\text{ cM}}_{{{\mathrm{coagulant}}}}$$

where a = electricity unit cost ($/KWh), b = the aluminum electrode’s cost ($/Kg), c = the coagulant cost ($/kg), and M_energy_, M_electrode_, M_coagulant_ = The amounts consumed throughout the procedure (in treatable m^3^ of wastewater).

## Results and discussion

### Implications of current density for urea removal

**Fig. 3:**
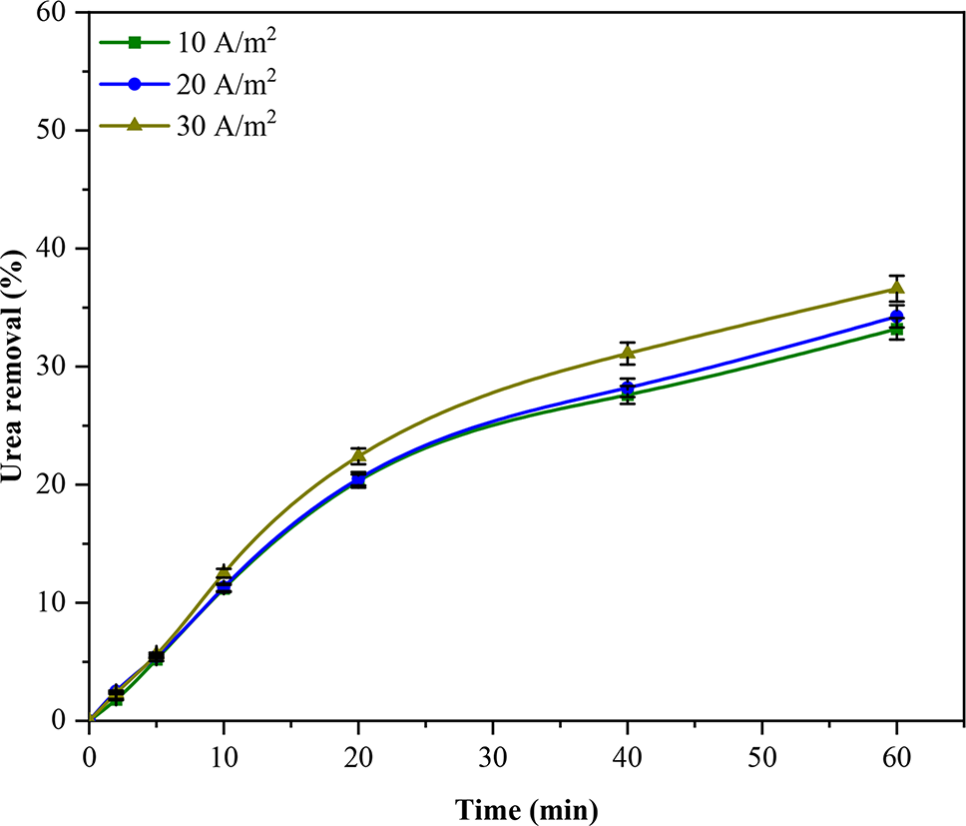
The influence of current density variation on urea removal (CD = 10, 20, 30 A/m^2^, urea conc. = 1000 mg/L, and time = 60 min).

Current density is a crucial EC parameter, the only directly controllable parameter in an EC cell. In an EC system, the density of the current dictates both the size and generation rate of H_2_ bubbles and metal hydroxide flocs, all of which can impact the efficacy of EC^30^. Figure 3 illustrates the influence of modifying the current density on the removal of urea from synthetic wastewater. The results indicate two distinct phases in which the percentage of urea removal increased with the duration of electrolysis. The rate of urea removal was rapid during the first 20 min of treatment. However, after the first 20 min, the rate of removal slowed. The growth in the percentage of urea removed during the first 20 min of the procedure can be ascribed to the anode’s dissolution and subsequent gas bubble formation, which were caused by the current applied to the anode^20,27^. The degree of urea removal is substantially impacted by the desorption phenomena that occur in the concluding phase of the process^21,22^. Furthermore, the electrocoagulation process may partially remove some organic compounds such as urea^31^. A marginal enhancement in urea removal efficiency was observed with the transition from 10 to 30 A/m^2^ of current density. This enhancement is attributed to the rise in the concentration of metal hydroxides, which can assimilate urea and consequently cause urea removal to increase^29^. After 60 min of exposure to 30 A/m^2^, the highest urea removal rate was recorded to be 36.60%. On account of the fact that the greatest amount of urea was extracted at a CD of 30 A/m^2^, subsequent experiments were conducted at that value.

### Inter-electrode spacing’s influence on EC

**Fig. 4:**
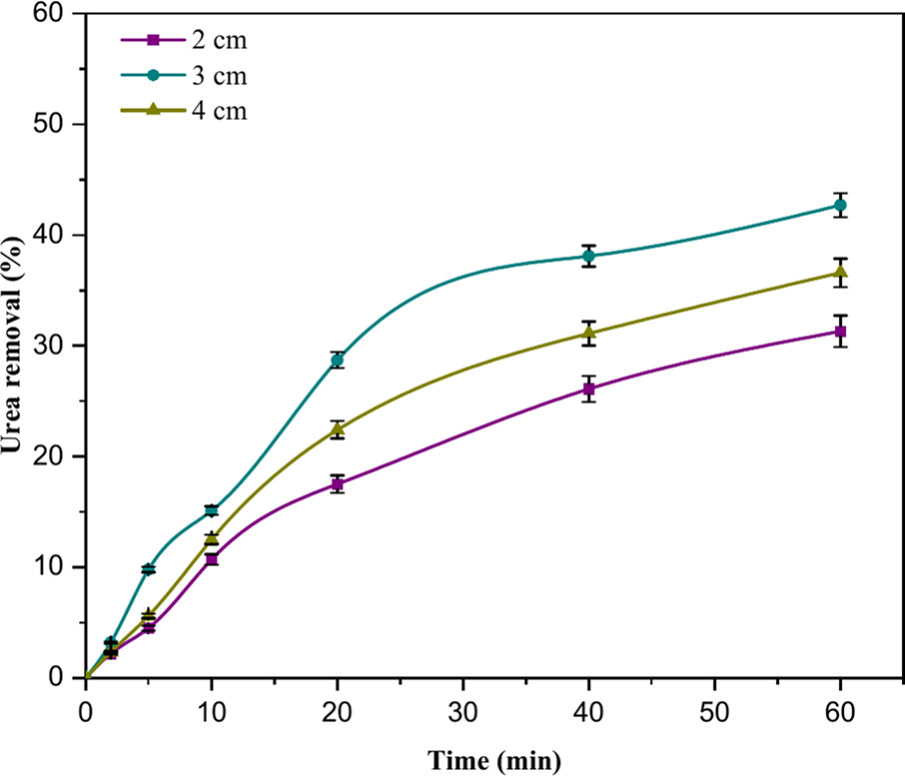
The influence of varying electrode spacing on urea removal (CD = 30 A/m^2^, urea conc. = 1000 mg/L, spacing = 2, 3, 4 cm, and time = 60 min).

The inter-electrode distance (IED) is a substantial determinant of EC efficacy, as the electrostatic field is contingent upon the separation between the anode and the cathode^32^. The experiments involved varying gap distances between electrodes (2, 3, and 4 cm) to determine the optimal spacing value within the investigated range. The initial urea concentration was maintained at 1000 mg/L, the initial pH was 7.80, the CD was set at 30 A/m^2^, and 0.5 g/L NaCl was used as the electrolyte. Based on the data presented in Fig. 4, it can be concluded that the urea removal efficiency rose from 31.30 to 42.70% when the interelectrode distance was 3 cm instead of 2 cm. The observed low removal effectiveness at a distance of 2 cm between the electrodes can be attributed to the deterioration of the formed aluminum hydroxide flocs due to collisions between them. Additionally, the high electrostatic attraction present in the system hinders appropriate mass transport for the electrocoagulation process^22,33^. At a distance of 3 cm between the electrodes, the urea removal efficiency reaches its maximum value of 42.70%. Potentially the most influential factor in the initiation of this phenomenon is the system configuration. By employing a circular reactor configuration, the gap between the walls of the beaker and the electrodes is equivalent to the 3 cm spacing between the electrodes. This correlation has an inherent relationship to the reactor’s cross-section. The consistency of the separation at the reactor indicates that (i) the distribution of the flocs is uniform. (ii) reducing the disruption of flocs that could potentially occur when a separation distance of 2 or 4 cm is utilised^27^. The efficacy of urea removal decreased from 42.70 to 36.60% when the electrodes’ distance apart changed from 3 to 4 cm. The decline in effectiveness was noted because the duration of ion travel extended as the distance increased, resulting in inadequate electrostatic attraction and consequently reduced floc formation, which is an essential for the coagulation of pollutants^20^.

### The effect of electrolyte type variation on EC

**Fig. 5:**
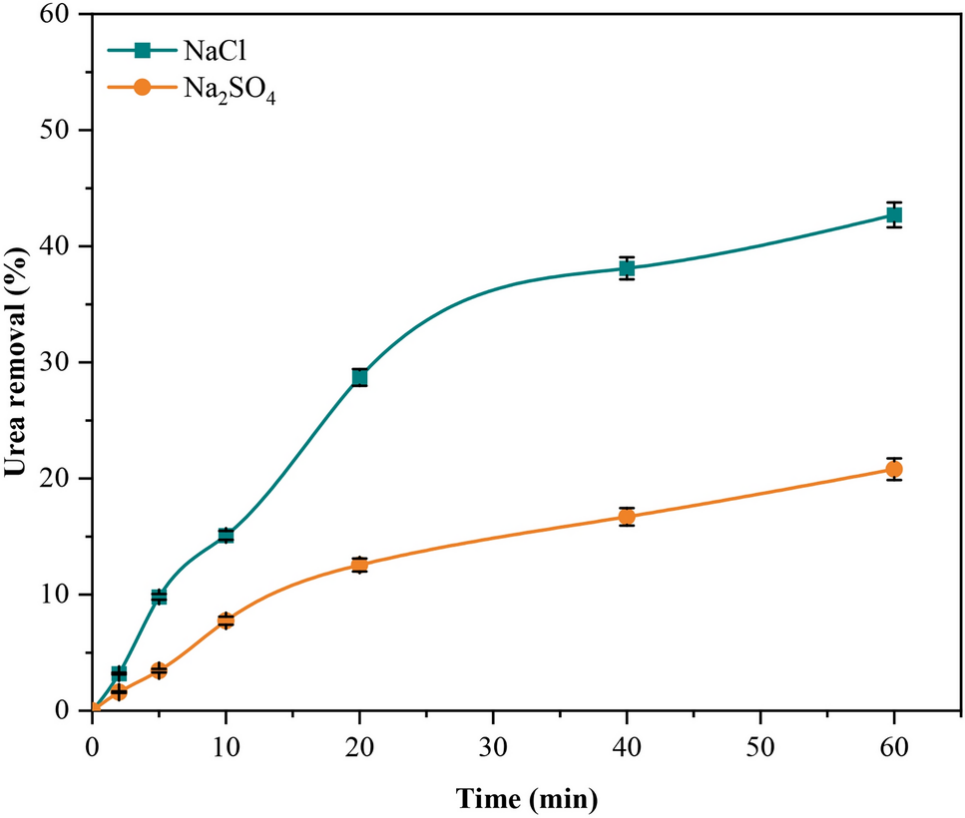
Electrolyte type’s impact on urea removal (CD = 30 A/m^2^, urea conc. = 1000 mg/L, utilised electrolytes (NaCl and Na_2_SO_4_), and time = 60 min).

The solution’s conductivity is a crucial component in the EC process^8^. The addition of an electrolyte to the solution, such as NaCl or Na_2_SO_4_, may result in an increase in conductivity, a drop in cell voltage at a fixed current density, and a reduction in electrical energy consumption^34^. Electrolyte type has a substantial impact on the efficacy of urea removal^22^. The supportive electrolytes utilised in the current investigation were NaCl and Na_2_SO_4_, which were selected owing to their accessibility and minimal toxicity^27,33^. The results of experiments conducted with NaCl and Na_2_SO_4_ at a constant concentration of 0.5 g/L were analysed and presented in Fig. 5. Research has indicated that Na_2_SO_4_ salt exhibits a reduced rate of urea removal in comparison to NaCl. The presence of SO_4_^−2^ ions in the supporting electrolyte is responsible for the reduced removal rate of urea in the presence of Na_2_SO_4_. These ions may form or strengthen a protective layer on the metal electrodes, thereby preventing localised corrosion of the aluminium electrodes^33,35^. Furthermore, EC process performance is negatively impacted when the current efficacy is decreased. The higher rate of urea removal achieved with the use of NaCl can be related to the presence of chloride ions (Cl^−^). These ions are capable of removing the passivation layer on the aluminium electrodes, resulting in increased generation of aluminium hydroxide through accelerated anodic dissolution^20,33^. Based on the findings of the experiment, it was shown that NaCl had an removal efficiency of 42.70% for urea while Na_2_SO_4_ had an efficiency of 20.80%.

### Response of EC to the variation of NaCl concentration and the electrolysis time

**Fig. 6:**
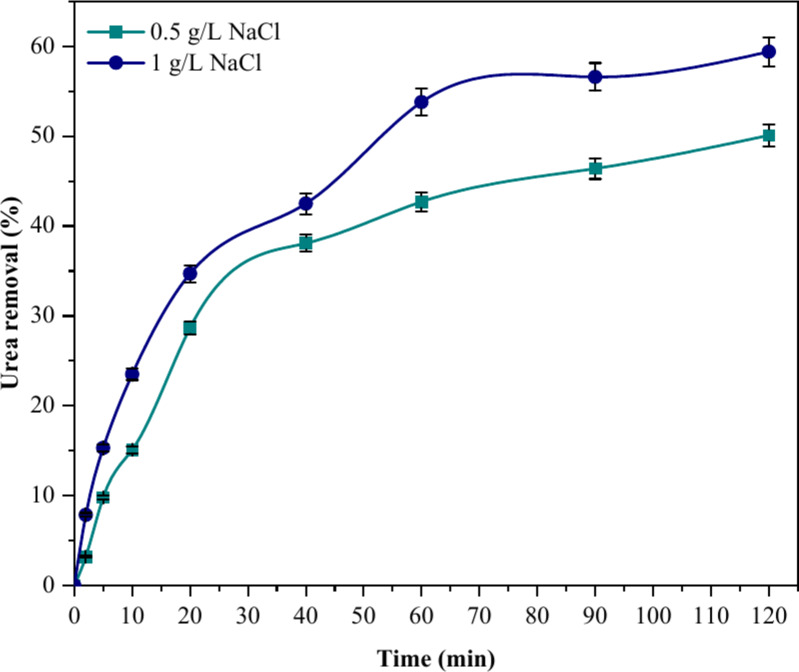
The influence of time and electrolyte concentration on urea removal (CD = 30 A/m^2^, urea conc. = 1000 mg/L, 0.5 and 1 g/L of NaCl as electrolyte concentration, and time = 120 min).

Incorporating electrolytes facilitates electrical transmission, leading to improved aluminum chemical dissolution^36^. The resistance between the electrodes is subsequently diminished as the ion concentration in the solution rises in response to an increase in salt concentration. The power consumption of electrolytic cells is diminished as the concentration of salt increases, while the cell voltage remains constant at a given current density^37^. The procedure of the experiment encompassed a 120-min investigation of the electrocoagulation process using two different concentrations of 0.5 and 1 g/L of NaCl salt at a current density of 30 A/m^2^, with the electrodes spaced 3 cm internally apart. It has been documented that the existence of chloride ions in solution can augment the efficiency of electrocoagulation processes by reducing the passivation of the aluminum surface^36^. An increase in NaCl concentration from 0.5 to 1 g/L results in greater metal dissolution, thereby improving the urea removal efficiency from 42.70 to 53.80% after 60 min of electrocoagulation treatment, as shown in Fig. 6. Despite the higher concentration of NaCl leading to increased urea removal, a concentration of 0.5 g/L of NaCl was selected for the EC-CC and CC-EC processes in synthetic wastewater, as it closely approximates the conductivity of domestic wastewater. Urea removal efficiency is significantly impacted by the duration of electrocoagulation treatment^27^. Time until the optimal duration of electrolysis is reached correlates positively with the removal efficacy. Nevertheless, beyond this point, the persistent presence of flocs ensures reliable removal efficiency^8,10^. A marginal enhancement in urea removal was observed with the addition of one hour to the treatment duration, especially with the higher concentration of the electrolyte. So, the treatment may be considered significant if it lasts for only one hour. This is consistent with earlier studies that show urea oxidation rate decreases with increasing electrolysis time^20,27^. In summary, the urea removal rate is increased by approximately 7.40% and 5.60%, respectively, when an additional 60 min of electrocoagulation treatment time is applied in the presence of 0.5 and 1 g/L of NaCl.

### The FTIR characterization of the by-products generated during EC.

**Fig. 7:**
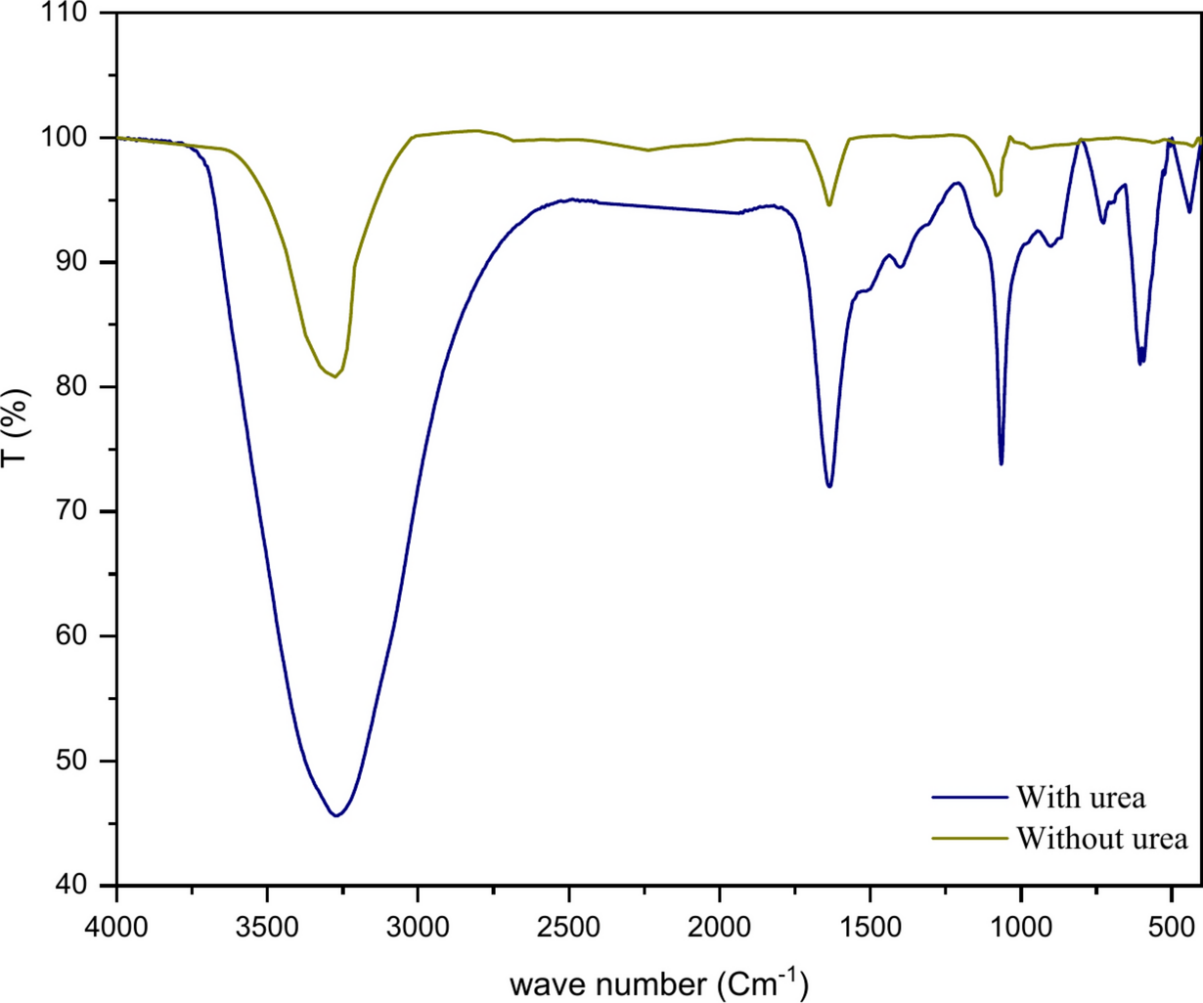
Analysis of the byproducts’ spectra using FTIR.

The by-products produced by the electrocoagulation process were analyzed and characterized using Fourier transform infrared spectroscopy (FTIR). The investigation involved two sludge samples, as shown in Fig. 7: one reference sample that contained only the electrolyte and another that contained both the urea and electrolyte. A filter paper was utilized to filter the sediment, which was then air-dried at room temperature for 24 h before FTIR analysis. Notable bands in the second sample sludge indicate the existence of groups associated with the urea compound. Both the 2800–3900 cm^–1^ and 1400–1800 cm^–1^ frequency bands are strongly indicative of the OH group’s presence, which ensures the occurrence of the adsorption of urea^20^. The additional urea compound groups, namely C–N, C=O, and N–H, are represented at 1465 cm^−1^, 1626 cm^−1^, and 3300–3400 cm^−1^, respectively^20,22,27^.

### Electrode morphology investigation:

**Fig. 8:**
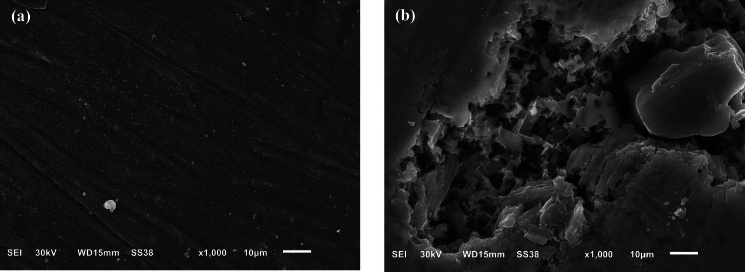
SEM images: (**a**) Al electrode prior to the EC process, and (**b**) Al electrode subsequent to the EC process.

Electrocoagulation induces corrosion, allowing the treatment’s occurrence. SEM images of the aluminum electrode prior to and subsequent to the electrocoagulation procedure, carried out at a current density of 30 A/m^2^, are depicted in Fig. 8. The abundance of cracks and voids on the anode surfaces is due to the depletion of minerals located on the active side, which is caused by the generation of oxygen at the surfaces of the electrodes. Irregular erosion can be characterized as the result of surface corrosion observed on the aluminum electrode^20^.

### Evaluation of the effectiveness of (EC-CC) and (CC-EC) in removing urea from synthetic wastewater:

**Fig. 9:**
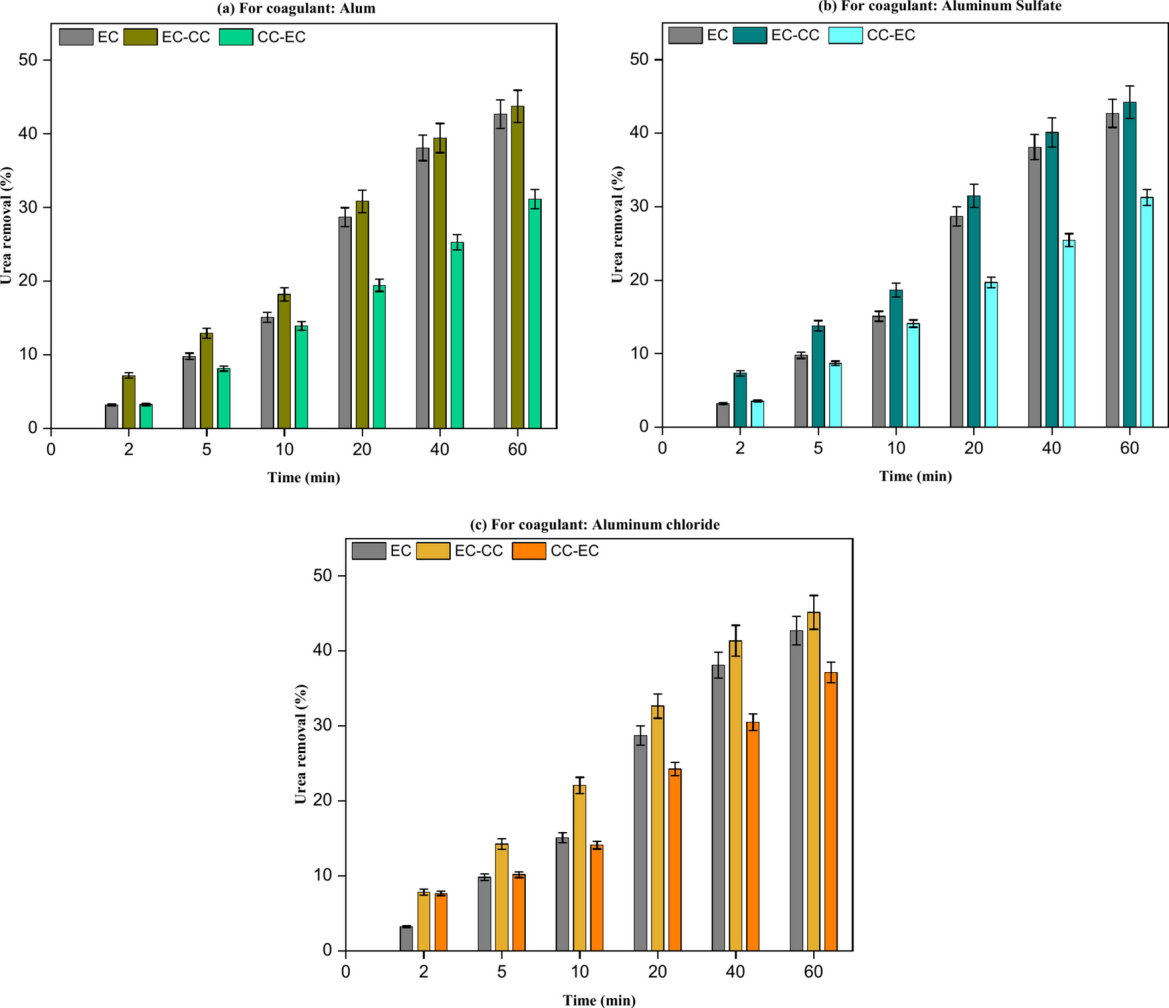
Evaluation of urea removal from synthetic wastewater (urea concentration = 1000 mg/L, pH = 7.80, and time = 60 min,) using EC alone, EC-CC, and CC-EC.

When conducting EC experiments under the following conditions, they were performed either prior to or subsequent to CC: Between the electrodes was a 3 cm distance, with CD = 30 A/m^2^ and NaCl concentration = 500 mg/L. As depicted in Fig. 9, the outcomes of EC-CC implementation revealed that urea removal from synthetic effluent did not improve significantly, regardless of the coagulant employed. The utilization of 0.5 g/L of alum, aluminum sulfate, and aluminum chloride was subsequent to the EC procedure, resulting in a slight improvement in the achieved results from 42.70 to 43.75%, 44.20%, and 45.15%, respectively. This minimal improvement in the rate of urea removal could be attributed to inadequate coagulant dose usage^27^. Not only that, but EC-CC and EC alone produced better results than the CC-EC technique. The decrease in urea removal rate utilizing CC-EC can be ascribed to the presence of SO_4_^–2^^27,33,35^. While the higher conductivity of aluminum chloride compared to alum and aluminum sulfate results in a higher removal urea for CC-EC process, but it is still lower than EC and EC-CC processes^38^. The maximum removal efficiency of urea using EC-CC, approximately 45%, surpasses the results obtained from earlier studies on urea removal through alternative processes like adsorption. According to prior research, the greatest urea removal effectiveness achieved by adsorption onto activated alumina was 24%^39^.

### EC-CC/CC-EC efficacy in the removal of urea and COD from real wastewater

**Fig. 10:**
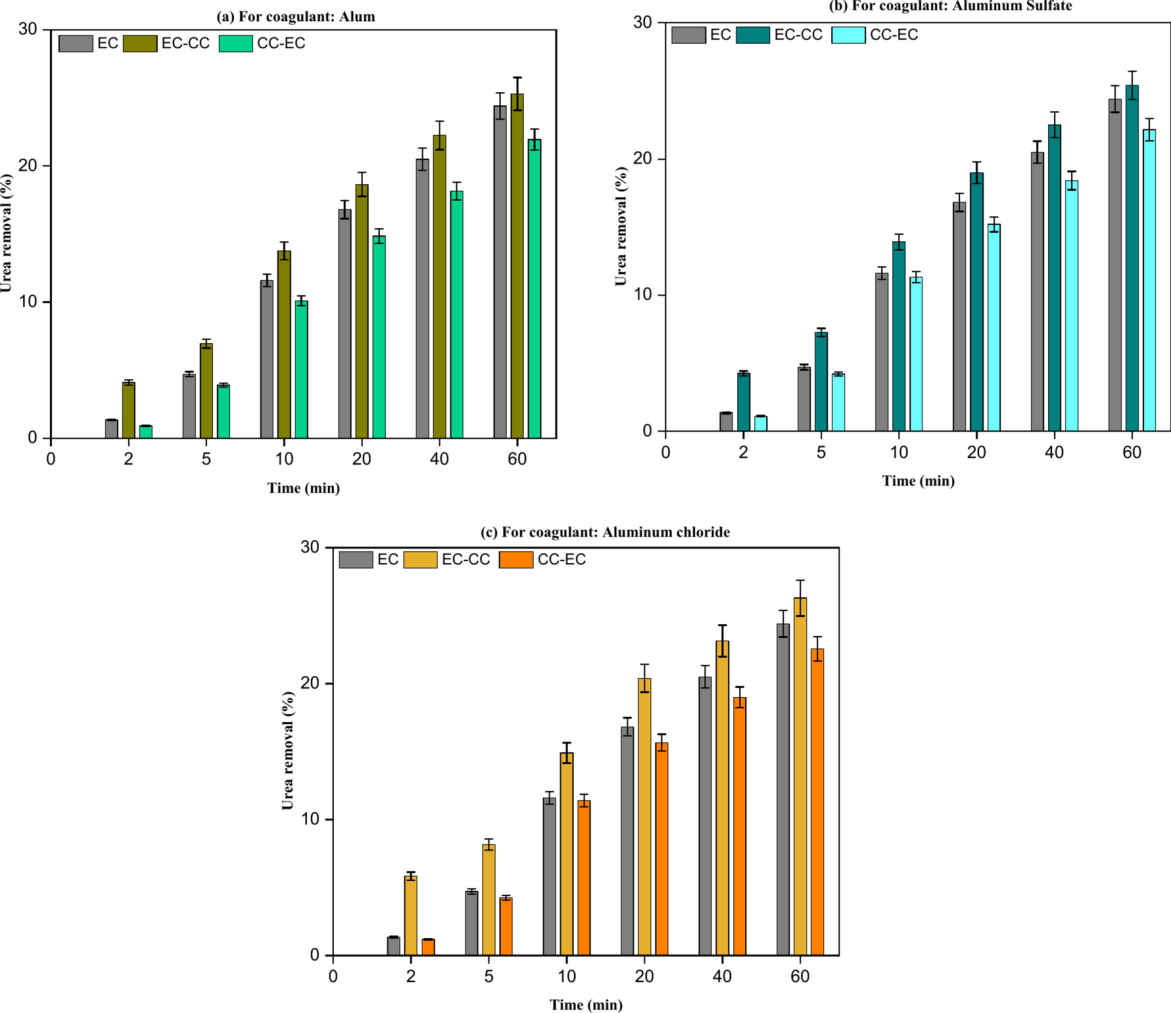
A comparison of urea removal from real effluent using EC alone, EC-CC, and CC-EC mechanisms: (Urea Conc. = 793 mg/L, pH = 7.80, and time = 60 min).

**Fig. 11:**
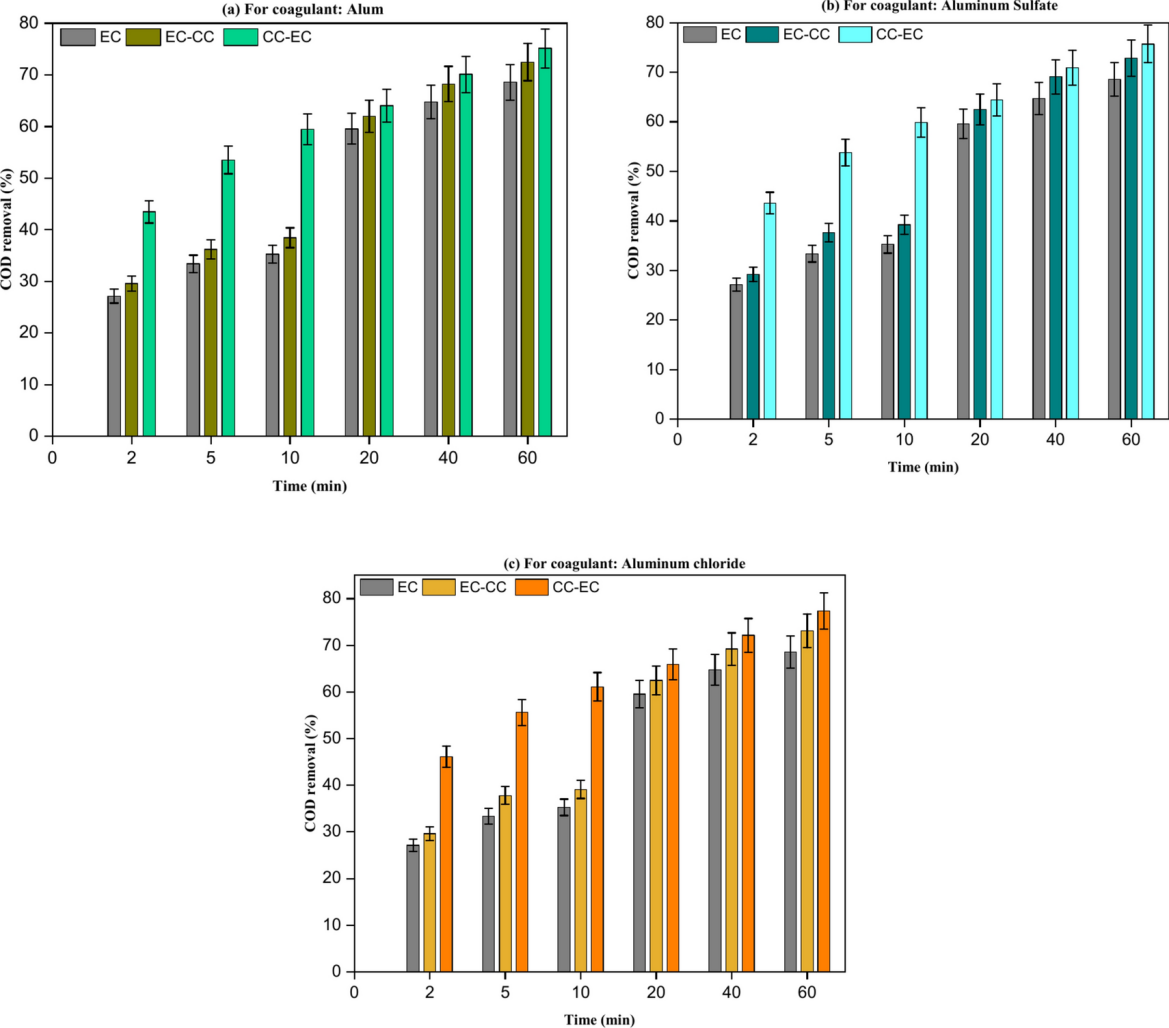
A comparison of the removal of COD from real effluent using EC alone, EC-CC, and CC-EC (COD = 560 mg/L, pH = 7.80, and time = 60 min).

The results of EC-CC implementation, as illustrated in Fig. 10, indicated that the implementation of any coagulant did not substantially enhance the removal of urea from real wastewater as well as synthetic wastewater. The result obtained from conducting EC alone is 24.40% for urea removal from real wastewater. The implementation of EC-CC treatment on real wastewater revealed a urea removal efficiency enhancement of approximately 0.90% for alum,1.00% for aluminum sulfate, and 1.89% for aluminum chloride. CC-EC has shown a decline in the rate of urea removal from real wastewater, and this decrease is attributed to the residual of SO_4_^–2^ in wastewater in case of using alum or aluminum sulfate after flocs sedimentation^33,35^. In the case of using aluminum chloride in the CC-EC process, the decline is attributed to the lower conductivity of the solution compared to the solution conductivity in the EC and EC-CC processes^27^. The optimal COD removal efficiency of 77.35% was attained by utilizing a CC with 0.5 g/L of aluminum chloride before conducting the EC procedure. The experiments conducted with CC-EC demonstrated a significant improvement in the removal performance of COD in comparison to the earlier runs utilizing EC and EC-CC as shown in Fig. 11. This demonstration is consistent with previous research efforts that sought to enhance the efficacy of COD removal from brewery effluent^24^.

### Economic evaluation

**Fig. 12:**
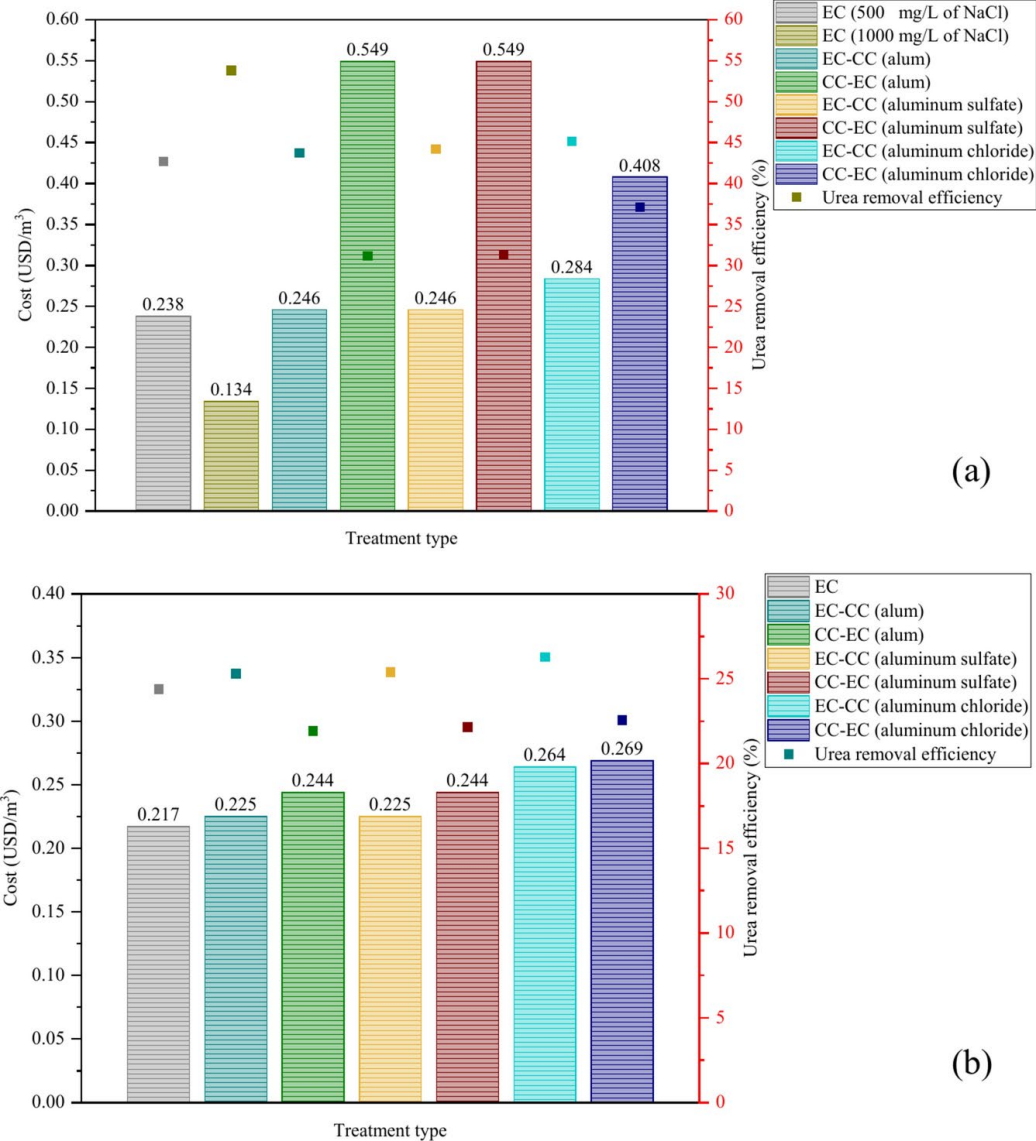
Operating cost comparison of EC, EC-CC, and CC-EC for urea removal from: (**a**) synthetic wastewater, and (**b**) real wastewater.

In addition to demonstrating enhanced efficacy in urea removal, the EC method incurs a marginally lower cost than the EC-CC or CC-EC procedures. Using alum and/or aluminum sulfate in the CC processes after the EC method does not significantly increase the cost of EC, as depicted in Fig. 12. Additionally, using aluminum chloride as the coagulant in the CC process after the EC process increased the cost of EC with higher value than using alum or aluminum sulfate. In contrast, the utilization of CC prior to EC significantly affects operational expenses, especially for synthetic wastewater. According to these findings, the most efficient and cost-effective method for urea removal from synthetic wastewater is EC, employing 1 g/L of NaCl as the electrolyte concentration. In the context of real wastewater, the electrocoagulation process for urea removal demonstrates the highest operational cost efficiency.

## Conclusion

- EC-CC may somewhat enhance the effectiveness of urea removal when utilizing alum, aluminum sulfate, and aluminum chloride in both synthetic and actual wastewater.
- CC-EC has a detrimental impact on urea removal efficiency when used with both synthetic and actual wastewater.
- The utilization of EC with an electrolyte concentration of 1000 mg/L demonstrated enhanced urea removal efficiency via synthetic wastewater and resulted in a maximal urea removal efficiency of 53.80%.
- CC-EC demonstrated a significant improvement in the effectiveness of COD removal from actual wastewater.
- EC only achieved 68.57% a COD removal efficiency, however CC-EC utilizing aluminum chloride as the coagulant achieved 77.35%.
- CC-EC achieved better removal of COD from the real wastewater than that achieved by EC-CC by about 4%.
- The cost-effectiveness of electrocoagulation was demonstrated through a comparison with combined chemical coagulation and electrocoagulation methods utilized for the removal of urea from real and synthetic effluent.

## Data Availability

All data generated or analysed during this study are included in this published article.

## Abbreviations

EC: Electrocoagulation
EC-CC: Electrocoagulation followed by chemical coagulation
CC-EC: Electrocoagulation following chemical coagulation
COD: Chemical oxygen demand
IED: Inter-electrode distance
